# Author Correction: Cellular senescence in white matter microglia is induced during ageing in mice and exacerbates the neuroinflammatory phenotype

**DOI:** 10.1038/s42003-025-09139-9

**Published:** 2025-11-06

**Authors:** Tatsuyuki Matsudaira, Sosuke Nakano, Yusuke Konishi, Shimpei Kawamoto, Ken Uemura, Tamae Kondo, Koki Sakurai, Takaaki Ozawa, Takatoshi Hikida, Okiru Komine, Koji Yamanaka, Yuki Fujita, Toshihide Yamashita, Tomonori Matsumoto, Eiji Hara

**Affiliations:** 1 Research Institute for Microbial Diseases (RIMD), Osaka University, 3-1 Yamadaoka, Suita, Osaka 565-0871 Japan; 2 Laboratory for Advanced Brain Functions, Institute for Protein Research, Osaka University, 3-2 Yamadaoka, Suita, Osaka 565-0871 Japan; 3 Department of Neuroscience and Pathobiology, Research Institute of Environmental Medicine, Nagoya University, Furo-cho, Chikusa-ku, Nagoya, 464-8601 Japan; 4 Department of Molecular Neuroscience, Graduate School of Medicine, Osaka University, 2-2 Yamadaoka, Suita, Osaka 565-0871 Japan; 5 Immunology Frontier Research Center (IFReC), Osaka University, 3-1 Yamadaoka, Suita, Osaka 565-0871 Japan; 6 Center for Infectious Diseases Education and Research (CiDER), Osaka University, 2-8 Yamadaoka, Suita, Osaka 565-0871 Japan

**Keywords:** Senescence, Microglia, Multiple sclerosis, Multiple sclerosis

Correction to: *Communications Biology* 10.1038/s42003-023-05027-2, published online 23 June 2023.

In the version of the article initially published, in Fig. 2h, the “Iba1” images for “Old” and “Young” were switched and have now been corrected in the HTML and PDF versions of the article. In Supplementary Data 1, the wrong source data was provided for Fig. 4a. The amended Supplementary Data 1 is now available alongside the online version of the article.

