# Supplementary material for: Cellular senescence in white matter microglia is induced during ageing in mice and exacerbates the neuroinflammatory phenotype
Source: Commun Biol. 2023 Jun 23;6:665. doi: 10.1038/s42003-023-05027-2 (PMC10290132; doi:10.1038/s42003-023-05027-2)
Supplement: Supplementary file 2 — Supplementary Information [file 42003_2023_5027_MOESM2_ESM.pdf]

Supplementary Fig. 1

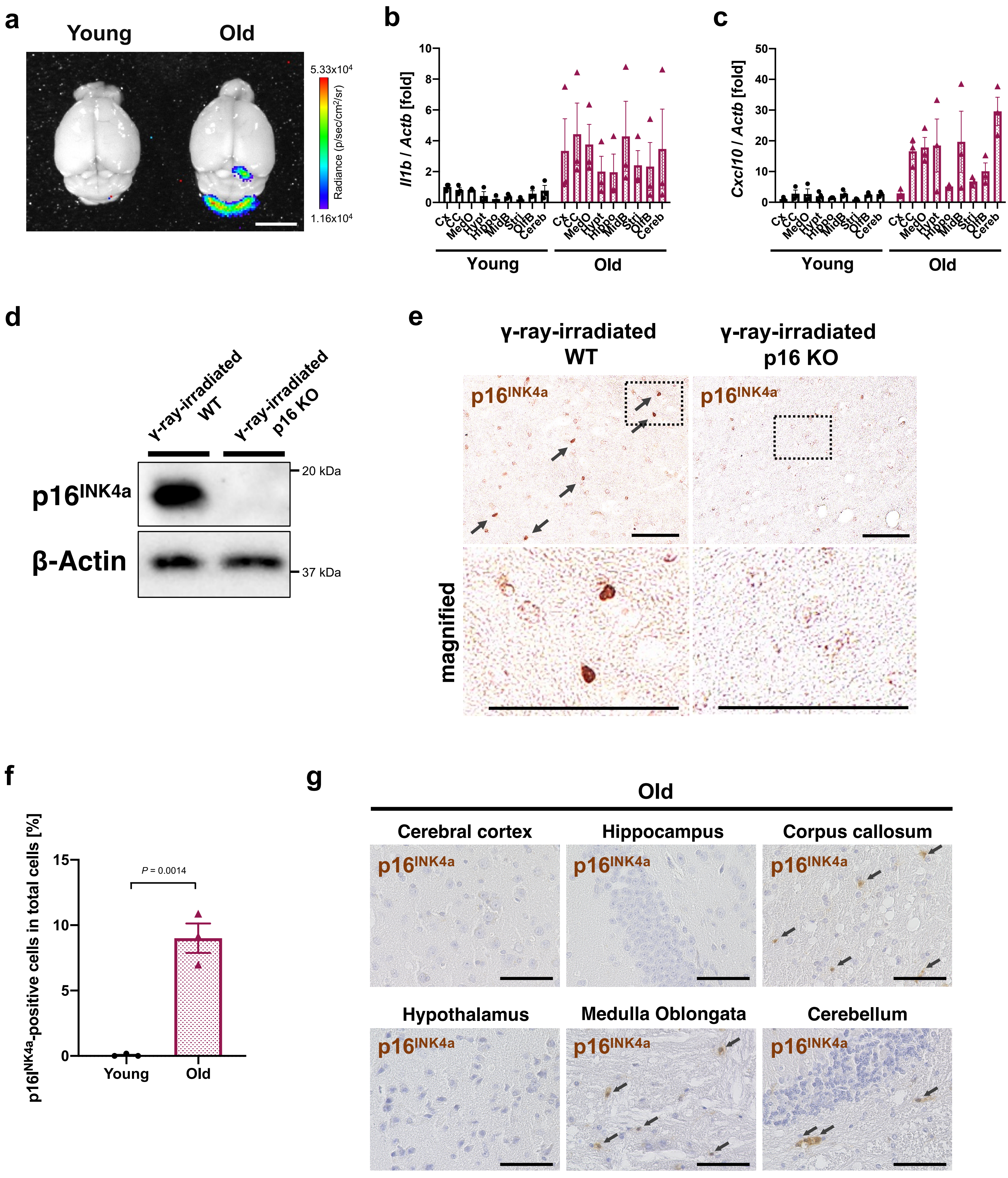

Supplementary Fig. 1

p16^{INK4a} positive cells accumulate in the white matter of old mice.

a Representative images showing the dorsal view of brains of female *p16-luc* mice (young: 2-3 months, old: 23-28 months) subjected to bioluminescence imaging. The colour bar indicates radiance values with minimum and maximum pixel thresholds. **b-c** RT-qPCR analysis of *Il1b* (**b**) or *Cxcl10* (**c**) mRNA expression in lysates of each brain region from young (2 months) or old (33 months) male mice. *Actb* was used as an internal control. **d** Lysates of Cd11b^{+} cells isolated by MACS from the whole brain of male WT (3 months) or p16 KO (3 months) mice irradiated with γ-ray at 10 Gy 3 months before sacrificing were immunoblotted for p16^{INK4a} protein. β-Actin was used as a loading control. **e** Immunohistochemistry of p16^{INK4a} protein in the medulla oblongata of male WT (3 months) or p16 KO (3 months) mice 3 months after irradiation with γ-rays. **f** Quantification of the number of p16^{INK4a}-positive cells as a percentage of total cells in corpus callosum of male WT mice - related to **main Fig. 1e** (young: 2 months, N = 3, n = 512 - 587 cells, old: 33 months, N = 3, n = 508 - 639 cells per mouse). **g** Immunohistochemistry of p16^{INK4a} protein in different regions of the old (28 months) male mouse brain. Nuclei were stained by hematoxylin. Data presented as mean ± S.E.M from three brains (for **b**, **c**). Arrows indicate p16^{INK4a}-positive cells. Scale bars, 5 mm (for **a**) or 50µm (for **e**, **g**). Abbreviations: Cx, cortex; CC, corpus callosum; MedO, medulla oblongata; Hypt, hypothalamus; Hippo, hippocampus; MidB, midbrain; Stri, striatum; OlfB, olfactory bulb; Cereb, cerebellum.

Supplementary Fig. 2

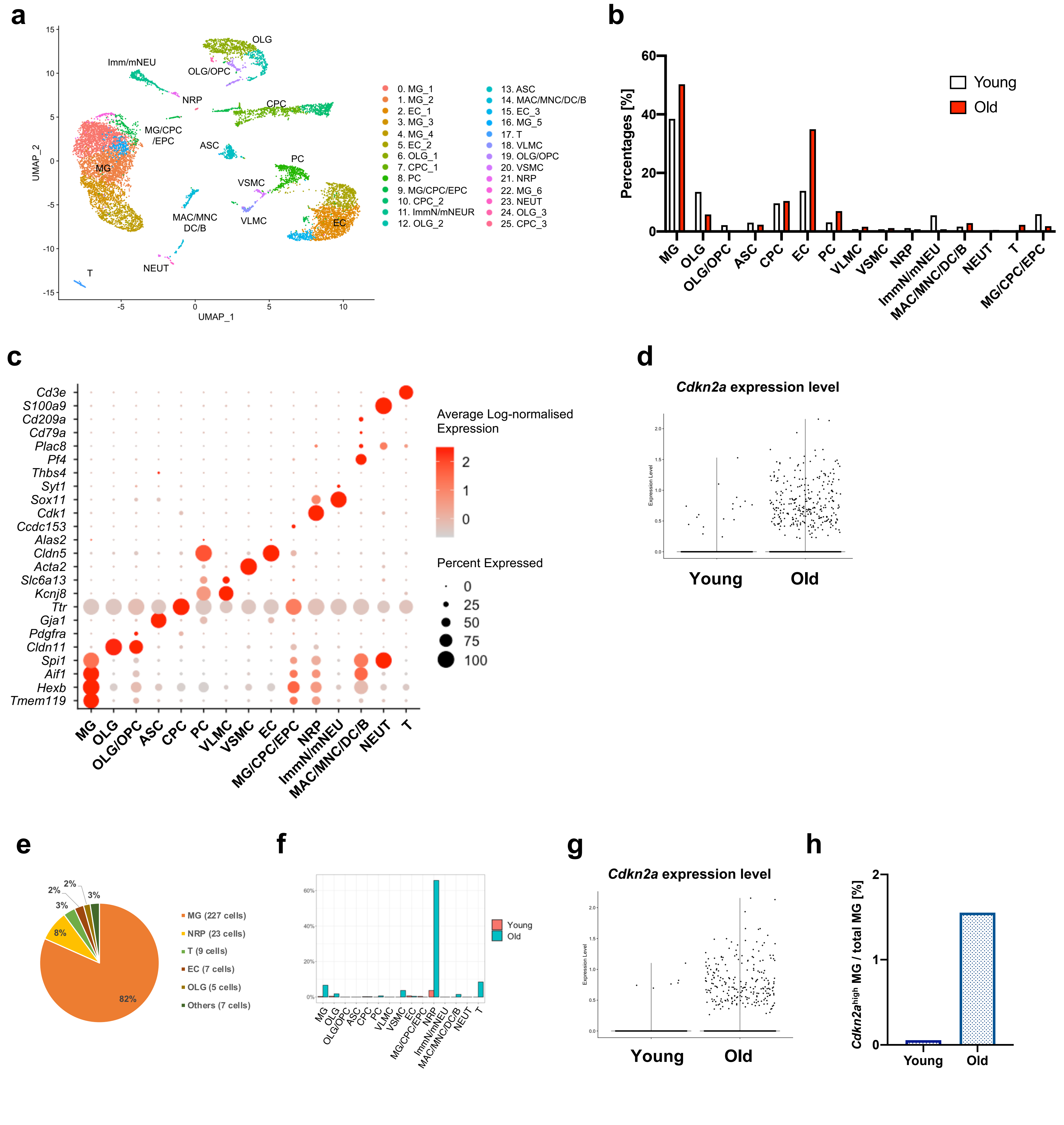

Supplementary Fig. 2

ScRNA-seq analysis of the corpus callosum in young and old mice.

a Single-cell RNA transcriptomic analysis of corpus callosum (CC) from young (2 months) or old (24 months) male mice. Uniform manifold approximation and projection (UMAP) plot of 11,446 cells (young: N = 1, n = 4,657 cells; old: N = 1, n = 6,789 cells) was analysed and clustered by Seurat. Each dot represents a single cell. **b** The percentages of each cell type in **main Fig. 2b** and **c**. **c** Bubble plot depicting the expression of cell type-specific genes in all sequenced cells from young and old CC for each cell type. **d** *Cdkn2a* expression in all corpus callosum cells from young or old mice as assessed by scRNA-seq. Each dot represents a single cell. **e** Proportion of *Cdkn2a*-expressing cells (expression level > 0; 278 cells in total) sorted by cell type in the old CC, related to (**d**). **f** Proportion of *Cdkn2a*-expressing cells (expression level > 0) in each cluster. **g** *Cdkn2a* expression levels in microglia in the corpus callosum of young or old mice assessed by scRNA-seq. Each dot represents a single cell. **h** Proportion of *Cdkn2a*-high (expression level ≧ 1) microglia (MG) in young and old mice, as a percentage of total MG (**g**). Abbreviations: oligodendrocyte precursor cells (OPC), oligodendrocytes (OLG), astrocytes (ASC), EPC (ependymocytes), neuronal-restricted precursors (NRP), immature neurons (ImmN), mature neurons (mNEU), choroid plexus epithelial cells (CPC), endothelial cells (EC), pericytes (PC), vascular smooth muscle cells (VSMC), vascular and leptomeningeal cells (VLMC), microglia (MG), monocytes (MNC), macrophages (MAC), T cells (T), neutrophils (NEUT), dendritic cells (DC) and B cells (B).

Supplementary Fig. 3

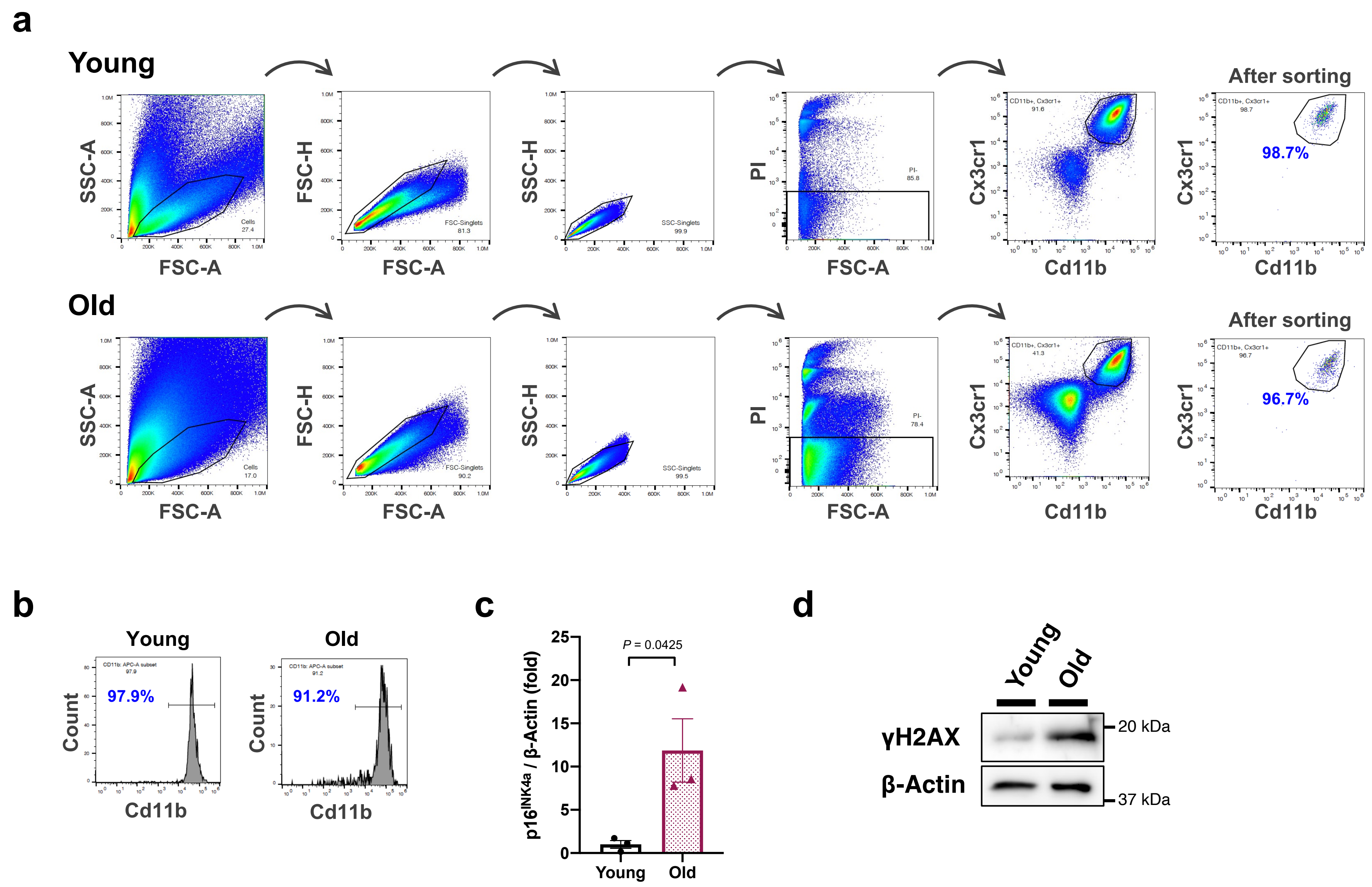

Supplementary Fig. 3

Sorting strategy for Cx3cr1^{+}/Cd11b^{+} microglia and supplementary figures to the main Fig. 2.

a Gating strategy for Cx3cr1^{+}/Cd11b^{+} microglia sorting from young or old mouse brains - related to **main Fig. 2f**. **b** Percentages of PI^{-} cells expressing Cd11b following MACS purification from young (2 months) or old (33 months) male mouse brains - related to **main Fig. 2g**. **c** Semi-quantification of the protein level of p16^{INK4a} in Cd11b-expressing cells purified by MACS from young (2 months) or old (32-33 months) male mouse brains - related to **main Fig. 2g**. **d** Lysates of MACS-isolated Cd11b^{+} cells from young (2 months) or old (33 months) male mouse brains were immunoblotted for γH2AX. β-Actin was used as a loading control. Data presented as mean ± S.E.M from three mice for **c**. Statistical significance was determined with two-tailed unpaired Student's t-test (**c**).

Supplementary Fig. 4

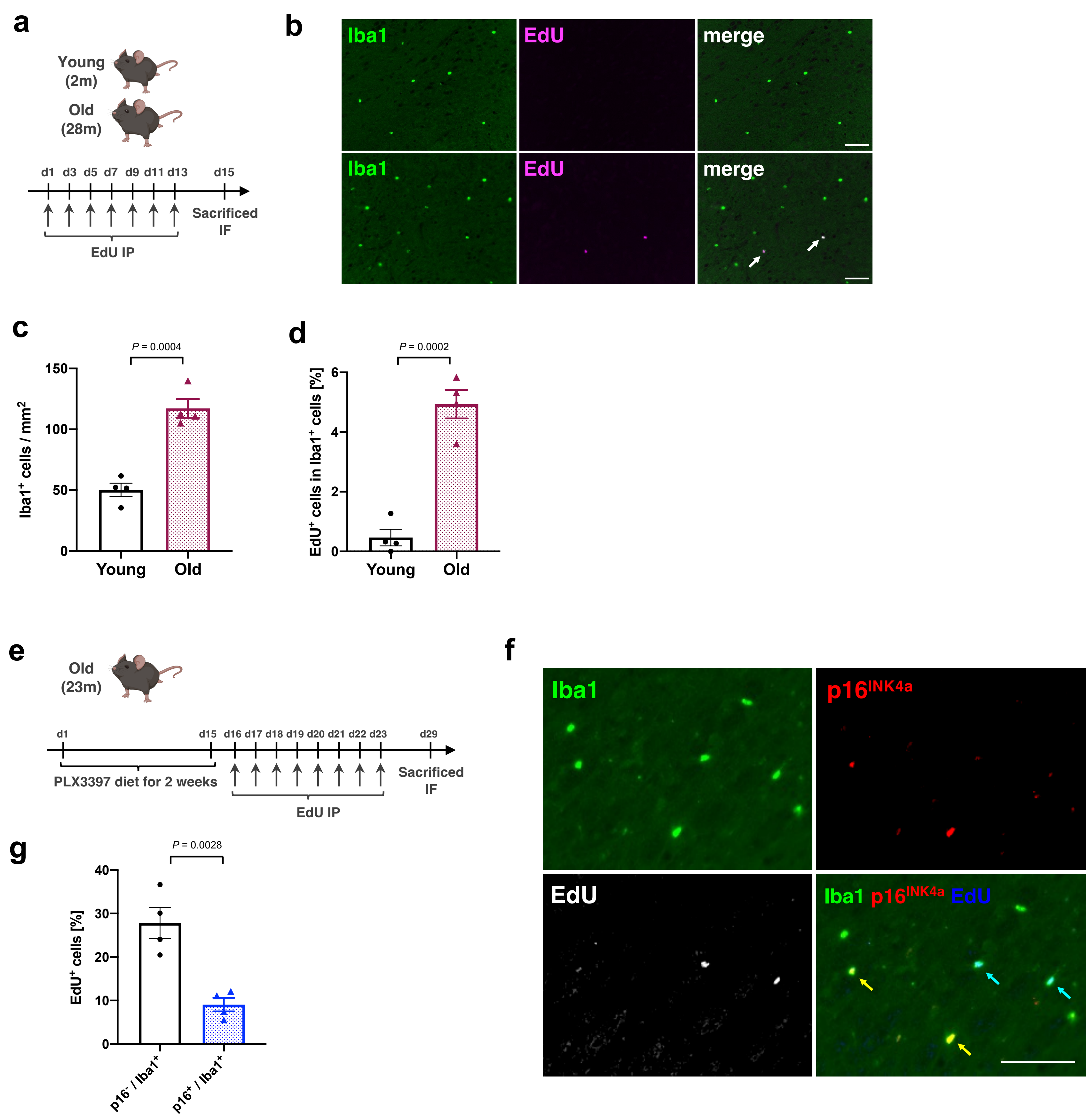

Supplementary Fig. 4

$p16^{INK4a}$ positive microglia are less proliferative.

a Overview of EdU treatment experiment in young (2 months, N = 4) or old (28 months, N = 4) male mice. EdU was intraperitoneally injected seven times in total for two weeks (on d1, d3, d5, d7, d9, d11, d13) and mice were then sacrificed on d15. **b** Representative immunofluorescence images for Iba1 in the medulla oblongata of young or old male mice, related to (**a**). EdU was detected using Click-iT EdU cell proliferation kit. **c-d** Quantification from (**b**) of Iba1 positive cells per mm^2 (**c**) and the number of EdU$^+$ microglia as a percentage of total Iba1$^+$ cells (**d**) (young: N = 4, n = 157 - 368 Iba1$^+$ cells, old: N = 4, n = 514 - 747 Iba1$^+$ cells per mouse). **e** Overview of PLX3397 and EdU treatment experiment in old (23 months, N = 4) male mice. Old mice were fed with PLX3397 diet for two weeks (on d1 to d15) and received daily intraperitoneal injections of EdU between d16 to d23. Mice were then sacrificed on d29. **f** Representative immunofluorescence images for Iba1 and $p16^{INK4a}$ in the medulla oblongata of old male mice which were fed with PLX3397 diet and injected with EdU, related to (**e**). Arrows indicate Iba1$^+$/p16$^+$/EdU$^-$ (yellow) or Iba1$^+$/p16$^-$/EdU$^+$ (sky blue) cells. **g** Percentages of EdU-positive cells amongst p16$^+$/Iba1$^+$ and p16$^-$/Iba1$^+$ cells in (**f**) (N = 4, n = 202 - 249 p16$^-$/Iba1$^+$ cells or 54 - 99 p16$^+$/Iba1$^+$ cells out of 301 - 325 Iba1$^+$ cells per mouse). The data represent mean ± S.E.M. Statistical significance was determined with two-tailed unpaired Student's t-test (**c**, **d**, **g**). Scale bar, 50 µm. The figure was created with BioRender.com (**a** and **e**).

Supplementary Fig. 5

a

Regulon	C0	C1	C2	C3	C4	C5	C6	C7	C8	C9	C10
Creb5_extended (25g)	1.30	2.38	0.62	1.70	1.02	0.55	0.29	0.99	0.40	2.03	0.20
Cebpa (11g)	1.37	1.94	0.79	1.61	1.21	0.93	0.41	1.19	0.26	1.00	0.52
Usf1_extended (18g)	1.28	1.91	0.65	1.61	1.20	0.96	0.32	1.07	0.30	1.15	0.77
Klf4_extended (81g)	1.23	1.89	0.71	1.46	1.04	0.79	0.51	1.05	0.44	1.47	0.60
Klf3_extended (203g)	1.24	1.85	0.66	1.56	1.12	0.84	0.27	1.04	0.16	1.89	0.67
Nfya (26g)	1.11	1.81	0.85	1.69	1.23	0.90	0.36	1.33	0.25	1.38	0.34
Fosb (35g)	1.34	1.76	0.86	1.51	1.24	1.01	0.43	1.11	0.30	1.12	0.51
Irf2 (11g)	1.23	1.74	0.79	1.44	1.11	0.91	0.42	1.03	0.26	1.44	0.80
Zbtb7a (18g)	0.95	1.69	0.58	1.42	1.03	0.66	0.18	1.28	0.10	3.31	0.46
Ikzf1_extended (440g)	1.30	1.56	0.87	1.36	1.22	1.07	0.54	1.06	0.37	1.06	0.72
Sox4 (17g)	1.37	1.52	0.96	1.30	1.26	1.17	0.65	1.02	0.39	0.67	0.82

b

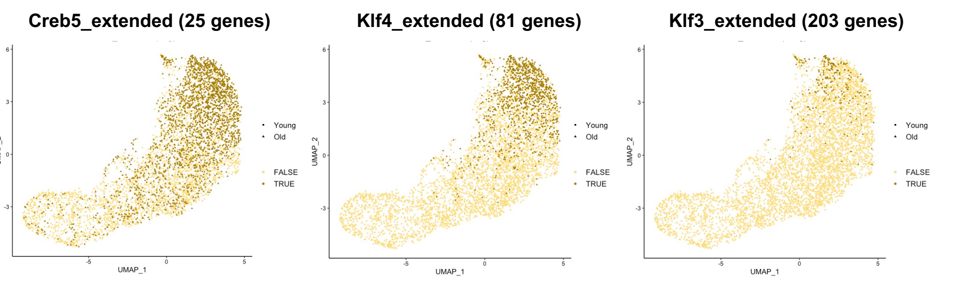

c

Regulon	C0	C1	C2	C3	C4	C5	C6	C7	C8	C9	C10
Stat2 (52g)	0.22	0.27	4.17	0.43	0.31	0.38	1.11	0.56	0.66	3.10	1.09
Irf9 (79g)	0.32	0.38	2.97	0.59	0.44	0.49	0.81	0.57	0.91	3.35	1.06
Maff (19g)	0.54	0.23	2.09	0.66	1.15	1.60	1.89	1.37	1.22	0.23	0.41
Bhlhe41 (11g)	0.28	0.27	1.99	0.60	0.49	0.81	1.65	1.42	1.11	1.02	1.69
Egr3_extended (41g)	0.77	0.46	1.80	1.09	1.47	1.70	1.04	1.67	0.45	0.44	0.39
Stat1 (101g)	0.56	0.50	1.77	0.61	0.67	0.81	2.02	0.76	2.03	0.56	1.04
Hivep2 (73g)	0.67	0.48	1.66	0.76	1.03	1.30	1.74	1.16	1.32	0.50	0.56
Bcl3 (47g)	0.73	0.50	1.57	0.82	1.14	1.38	1.56	1.20	1.36	0.50	0.41
Irf7 (131g)	0.60	0.54	1.53	0.65	0.72	0.86	1.86	0.86	1.93	0.63	1.03
Batf (14g)	0.81	0.64	1.42	0.95	1.20	1.42	1.24	1.08	1.13	0.97	0.27
Fosl2 (52g)	0.42	0.42	1.34	0.66	0.68	0.84	2.10	1.04	3.08	0.57	0.42

d

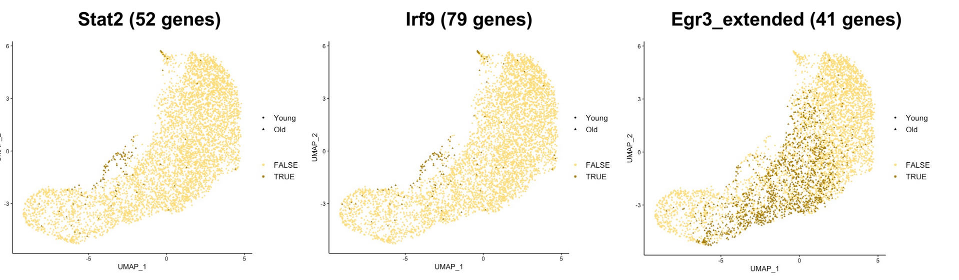

e

Regulon	C0	C1	C2	C3	C4	C5	C6	C7	C8	C9	C10
Cpeb1_extended (25g)	0.01	0.01	0.14	0.01	0.01	0.02	3.00	0.10	24.42	0.16	0.14
Nfil3 (20g)	0.12	0.12	0.72	0.35	0.21	0.25	2.28	0.70	9.42	0.51	0.49
Zfp362_extended (18g)	0.23	0.55	0.14	0.55	0.36	0.31	0.55	0.28	3.88	4.84	1.24
Klf9_extended (471g)	0.28	0.35	0.51	0.45	0.26	0.29	1.65	0.61	3.70	3.09	0.99
Prdm1_extended (351g)	0.44	0.38	1.19	0.52	0.54	0.71	2.19	0.85	3.27	0.73	0.82
Fosl2 (52g)	0.42	0.42	1.34	0.66	0.68	0.84	2.10	1.04	3.08	0.57	0.42
Hif1a (414g)	0.63	0.56	0.99	0.66	0.67	0.76	1.71	0.85	2.40	0.99	1.04
Rxrg (13g)	0.14	0.09	1.31	0.29	0.27	0.30	3.18	0.64	2.14	2.06	1.48
Stat1 (101g)	0.56	0.50	1.77	0.61	0.67	0.81	2.02	0.76	2.03	0.56	1.04
Irf7 (131g)	0.60	0.54	1.53	0.65	0.72	0.86	1.86	0.86	1.93	0.63	1.03

f

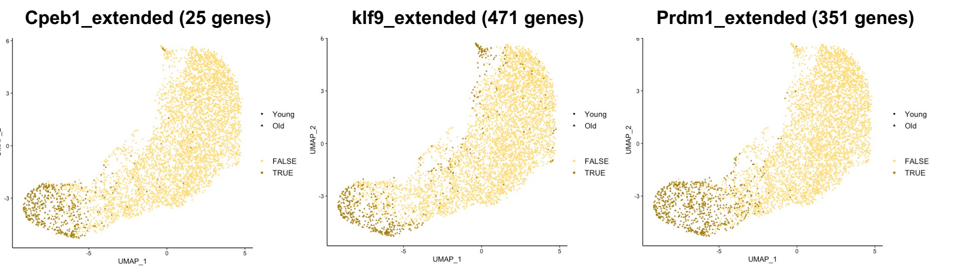

g

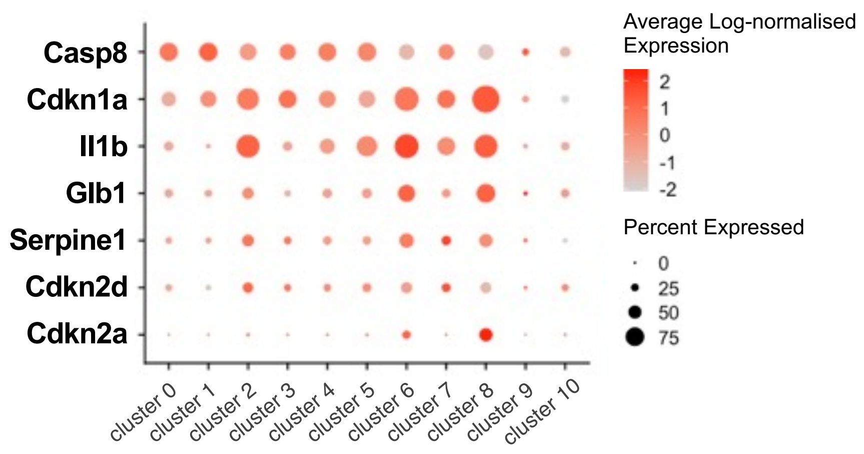

h

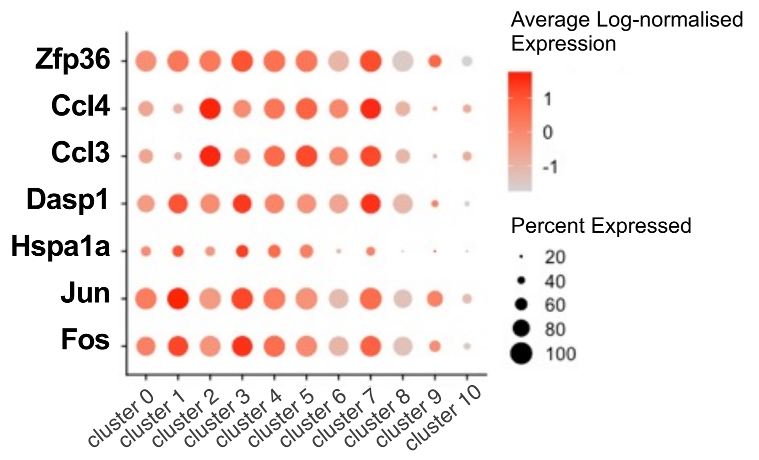

i

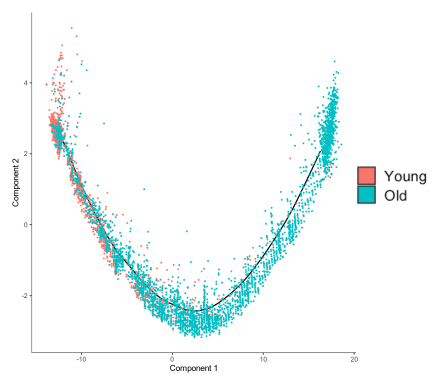

j

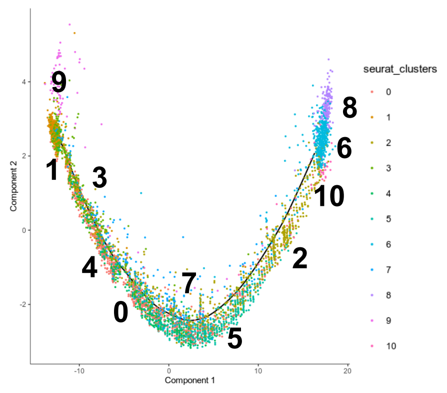

k

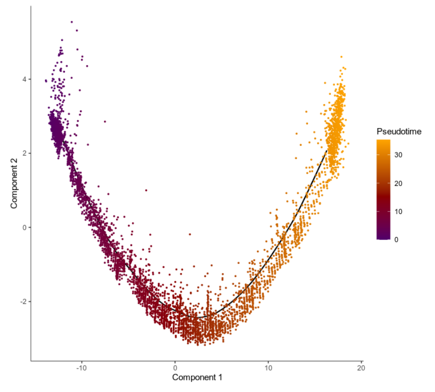

l

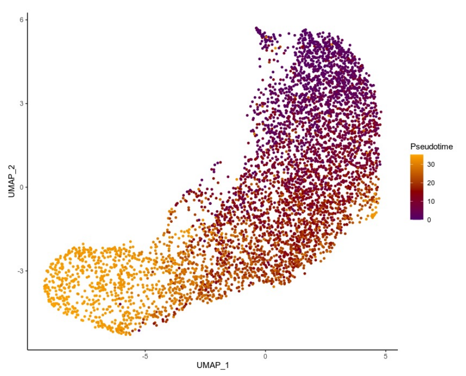

Supplementary Fig. 5

SCENIC, gene expression and Monocle analysis of scRNA-seq in young and old white matter microglia.

a-f Regulon score analysed by SCENIC. Top 10 valuable genes and relative regulon scores in cluster 1 (**a**), cluster 2 (**c**) and cluster 8 (**e**) are shown. Representative SCENIC UMAP binary plots of genes regulated by each regulon in cluster 1 (**b**), cluster 2 (**d**) and cluster 8 (**f**) are also depicted. The colours in the cells are defined by the maximum (red), median (yellow) and minimum (green) of the percentiles in each column. **g-h** Bubble plot depicting the expression of senescnece signature genes (**g**) or exAM-related genes (**h**) in microglia obtained from young and old CC for each microglial subcluster (clusters 1-10). **i-l** Monocle analysis of white matter microglia in young and old mice. Classification by age (**i**), cluster (**j**), pseudotime (**k**) or pseudotime of UMAP plot (**l**) was shown. Each dot represents a single microglia.

Supplementary Fig. 6

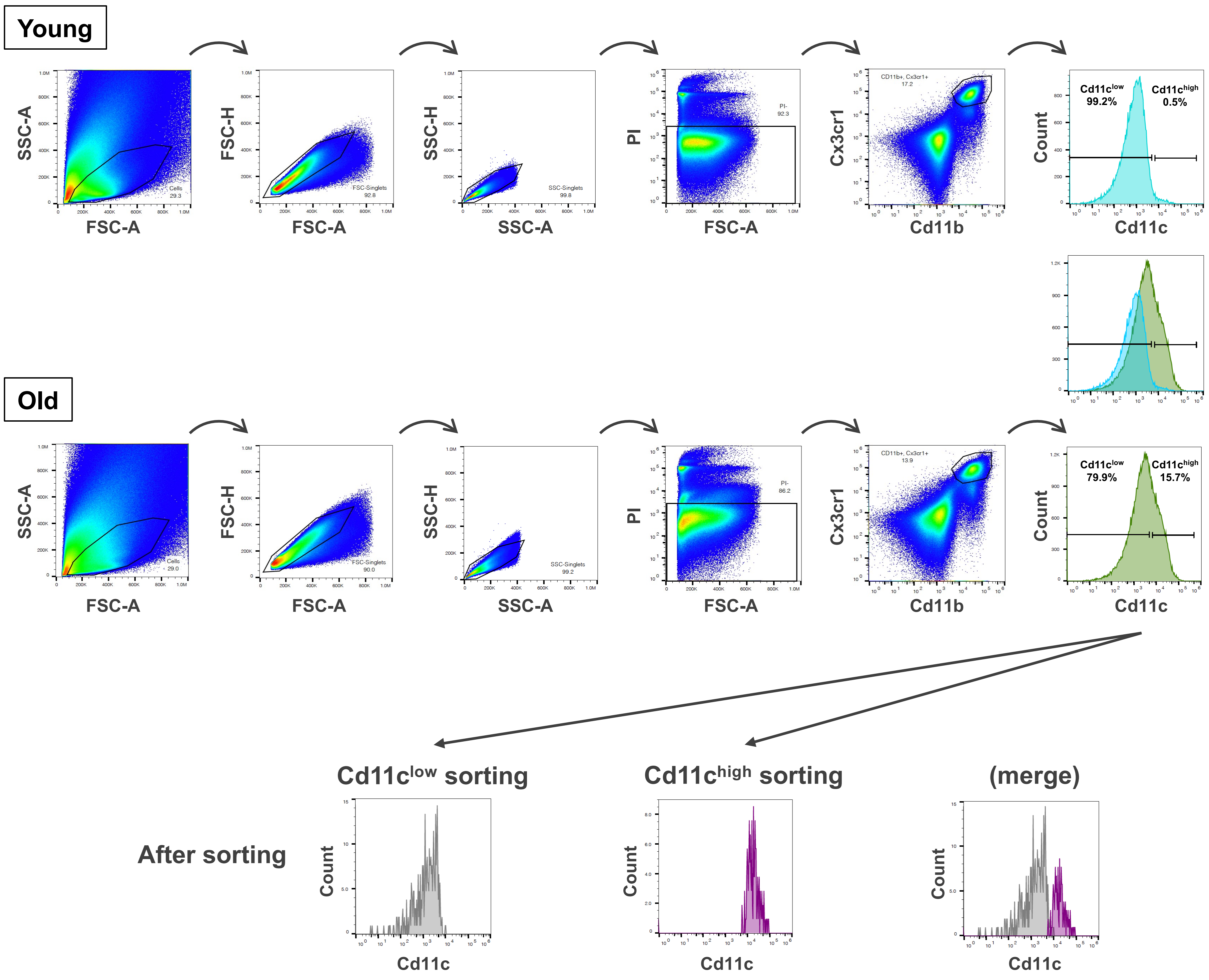

Supplementary Fig. 6

Sorting strategy for Cd11c^{low} or Cd11c^{high} microglia.

Gating strategy for Cd11c^{low} or Cd11c^{high} microglia (Cx3cr1^{+}/Cd11b^{+}) sorting from old mouse brains, related to **main Fig. 3i**.

The threshold of Cd11c-high and -low was determined from microglia in young (6 months) and old (28-29 months) male mice. The purity of Cd11c^{low} or Cd11c^{high} cells by using one-sixtieth of the solution after sorting was examined.

Supplementary Fig. 7

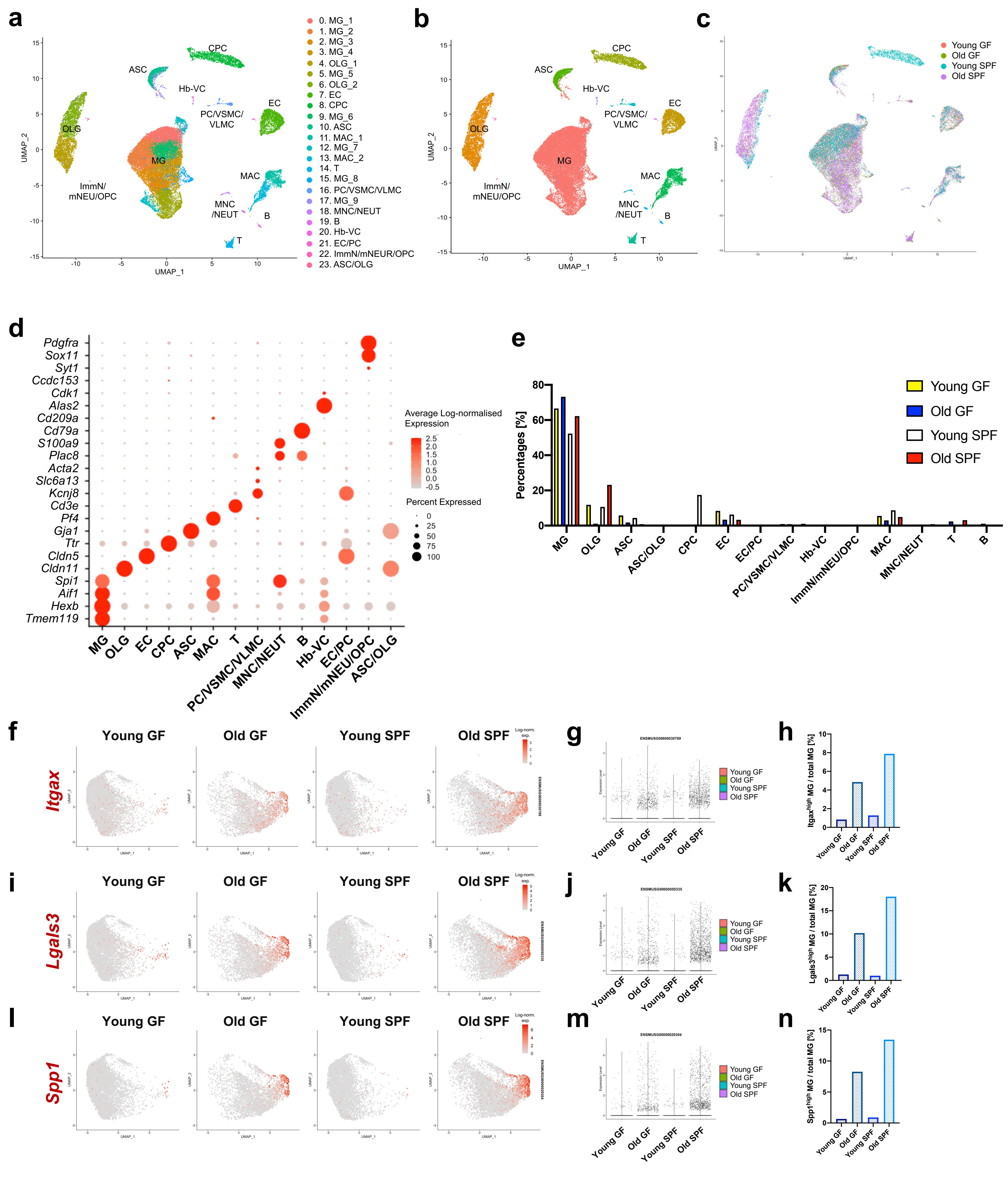

Supplementary Fig. 7

ScRNA-seq analysis of young GF, young SPF old GF and old SPF mice.

a Single-cell RNA transcriptomic analysis of medulla oblongata from young GF, young SPF, old GF and old SPF male mice. UMAP plot of 30,854 cells (young GF (3 months), N = 1, n = 8,650 cells; old GF (25 months), N = 1, n = 4,715 cells, young SPF (3 months), N = 1, n = 8,787 cells; old SPF (23 months), N = 1, n = 8,702 cells) was analysed by Seurat. Each dot represents a single cell. **b** UMAP plot in (**a**) was annotated by each cell type using cell type-specific markers. Each dot represents a single cell. **c** UMAP plot of cells annotated by each sample (Young GF, red; Old GF, green; Young SPF, blue; Old SPF, purple) **d** Bubble plot depicting the expression of cell type-specific genes amongst populations shown in (**b**). **e** Cell type distribution of each sample type in (**b** and **c**). **f-n** UMAP plots depicting the expression patterns of *Itgax* (**f**), *Lgals3* (**i**) and *Spp1* (**l**) genes in microglia from each sample type. Each dot represents a single microglia. Overall single-cell expression levels of *Itgax* (**g**), *Lgals3* (**j**) and *Spp1* (**m**) are shown. Percentages of microglia highly expressing (expression level ≧ 1) *Itgax* (**h**), *Lgals3* (**k**) and *Spp1* (**n**) are also shown. Log-norm. exp., Log-normalised expression. Abbreviations: oligodendrocyte precursor cells (OPC), oligodendrocytes (OLG), astrocytes (ASC), EPC (ependymocytes), neuronal-restricted precursors (NRP), immature neurons (ImmN), mature neurons (mNEU), choroid plexus epithelial cells (CPC), endothelial cells (EC), pericytes (PC), vascular smooth muscle cells (VSMC), hemoglobin-expressing vascular cells (Hb-VC), vascular and leptomeningeal cells (VLMC), microglia (MG), monocytes (MNC), macrophages (MAC), T cells (T), neutrophils (NEUT), dendritic cells (DC) and B cells (B).

Supplementary Fig. 8

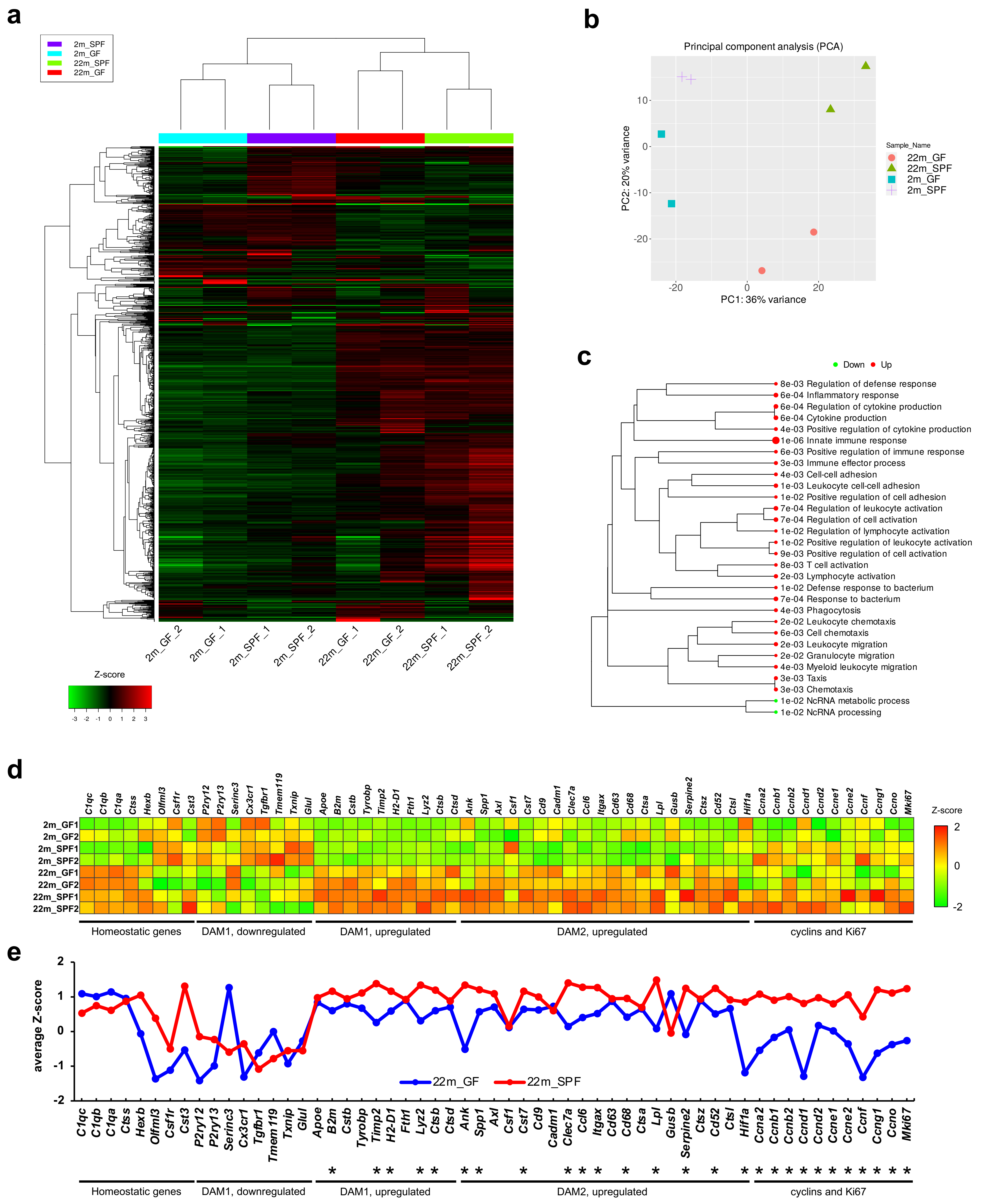

Supplementary Fig. 8

Bulk RNA-seq analysis of young GF, young SPF, old GF and old SPF mice.

a Heatmap showing expression patterns of the top 1000 most variable genes amongst microglia (Cd45^{low}, Cd11b^{+} cells) sorted by FACS from young GF (2 months, N = 2), young SPF (2 months, N = 2), old GF (22 months, N = 2) or old SPF (22 months, N = 2) male mice, analysed with iDEP. **b** Principal component analysis (PCA) of the RNA-seq data. **c** The gene ontology (biological process) pathway tree for comparison between old SPF and old GF mice was analysed using the GAGE method in iDEP. Both up-regulated pathways (red) and down-regulated pathways (green) in old SPF mice compared to old GF mice were mapped. **d** Bulk RNA-seq of whole brain microglia in young SPF, young GF, old SPF and old GF mice. Genes associated with homeostatic state, DAM state 1 (DAM1)-related genes, DAM2-related genes and genes associated with cyclins and Ki67 are depicted. Scale, Z-score from -2 to 2. **e** The graph depicts the average of Z-score of each gene in old SPF/GF mice. Genes highly expressed in 22m_SPF ([Z-score (22m_SPF)] - [Z-score (22m_GF)] > 0.5) are marked with asterisks.

Supplementary Fig. 9

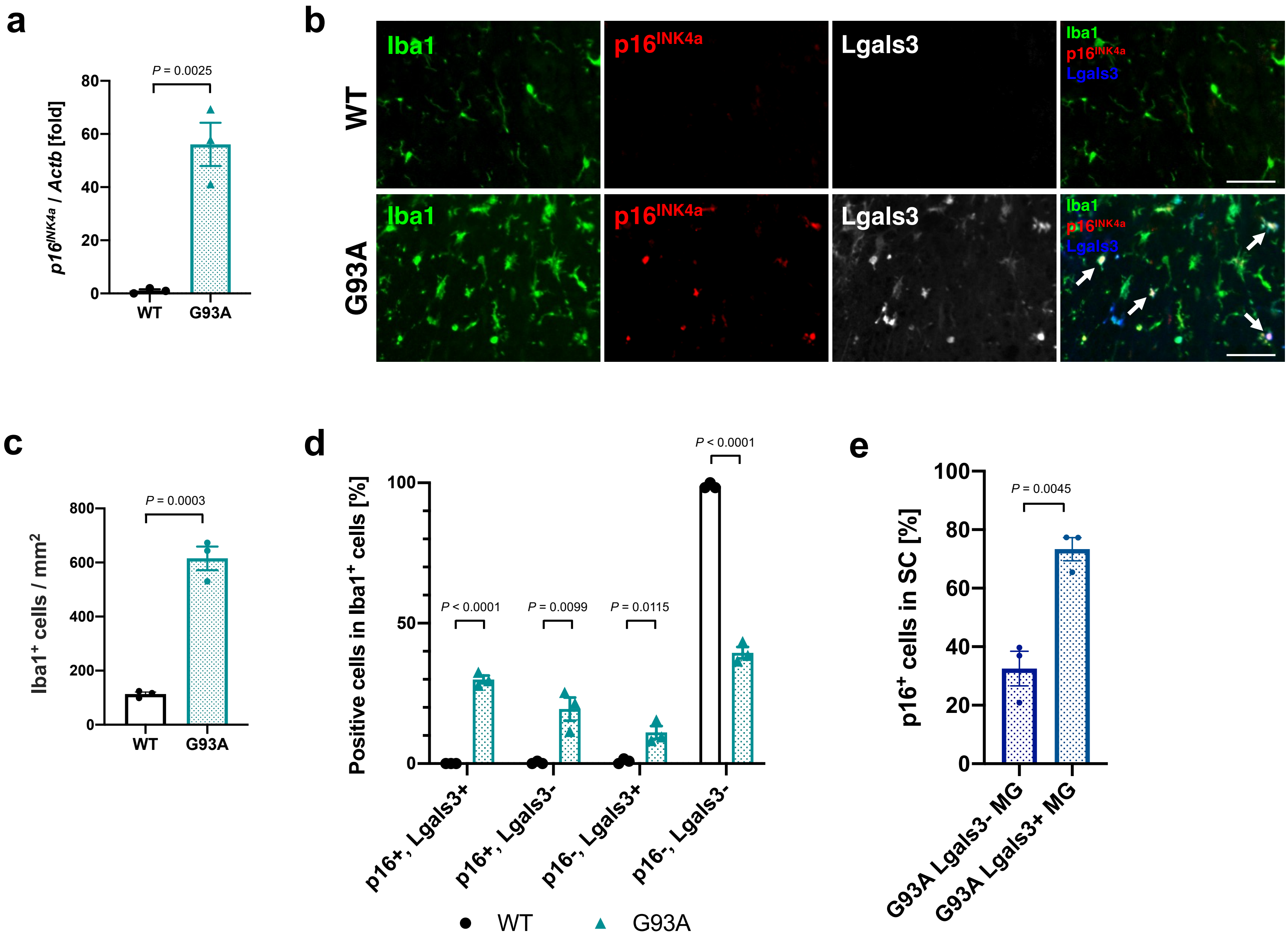

Supplementary Fig. 9

p16^{INK4a} positive senescent microglia accumulate in the spinal cord of paralytic SOD1 G93A mice.

a RT-qPCR analysis of *p16^{INK4a}* mRNA expression in spinal cord lysates from male WT (5 months, N = 3) or SOD1 G93A (5 months, N = 3) mice. *Actb* was used as an internal control. **b** Representative immunofluorescence images for Iba1 (green), p16^{INK4a} (red) and Lgals3 (white or blue) in the spinal cord of WT or SOD1 G93A mice. Arrows indicate triple-positive (Iba1$^+$/p16$^+$/Lgals3$^+$) cells. **c** Density of Iba1$^+$ cells in the spinal cord of WT (5-6 months, N = 3, n = 113 - 119 Iba1$^+$ cells per mouse) or SOD1 G93A (5-6 months, N = 3, n = 122 - 126 Iba1$^+$ cells per mouse). **d** Quantification of p16$^+$/Lgals3$^+$, p16$^+$/Lgals3$^-$, p16$^-$/Lgals3$^+$ and p16$^-$/Lgals3$^-$ Iba1-positive cells in (**b**). **e** Percentage of Iba1$^+$/Lgals3$^-$ or Iba1$^+$/Lgals3$^+$ cells from (**d**) expressing p16^{INK4a} in SOD1 G93A mice. Data presented as mean ± S.E.M. Statistical significance was determined with two-tailed unpaired Student's t-test (**a**, **c**, **d**, **e**). Scale bar, 50 µm. MG; microglia.

Supplementary Fig. 10

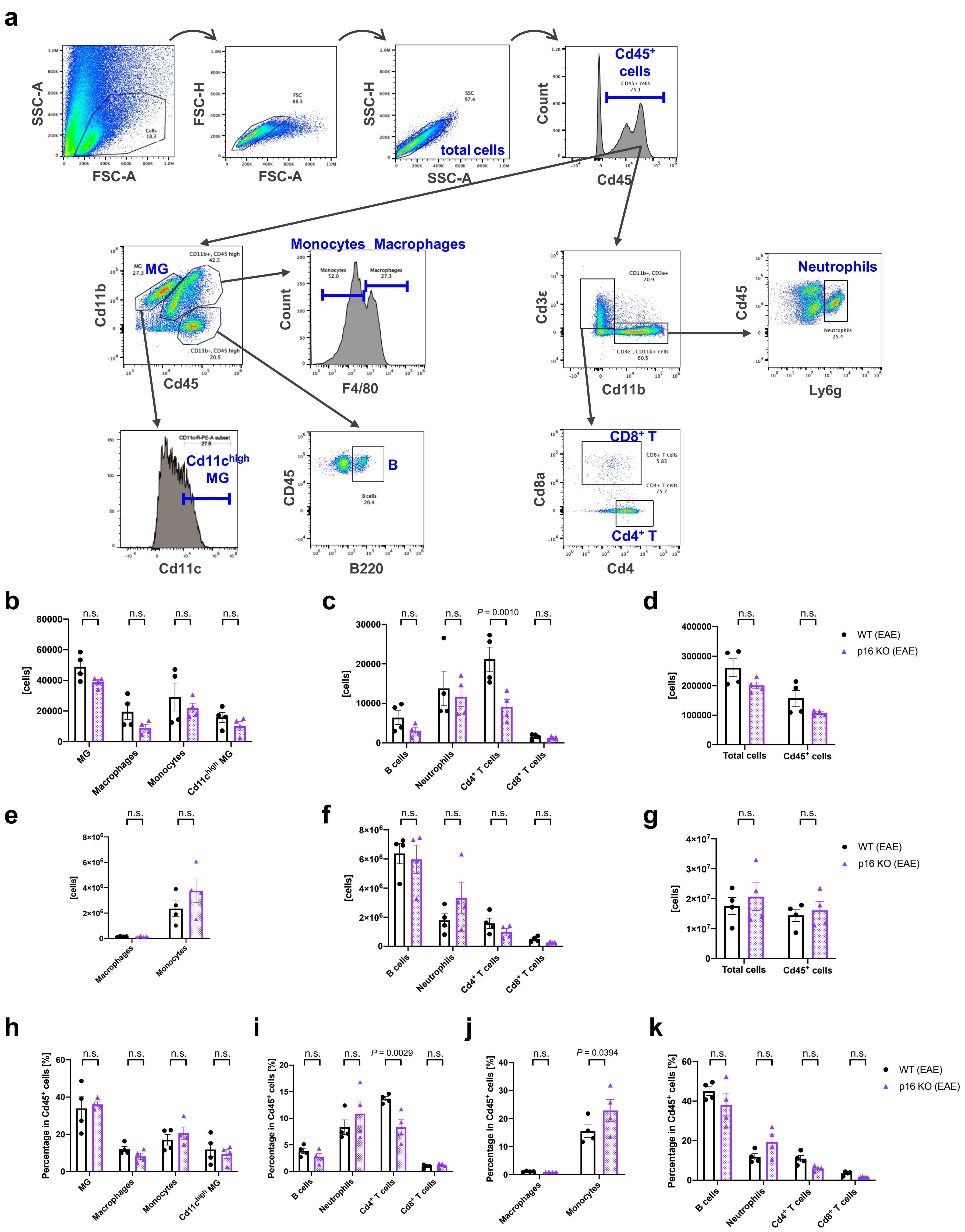

Supplementary Fig. 10
p16[INK4a] knockout decreases T cell infiltration in the spinal cord of EAE-induced mice.
a Gating strategy for immune cells (microglia (MG), macrophages, monocytes, Cd11c^{high} MG, B cells, neutrophils, Cd4$^+$ T cells, Cd8$^+$ T cells and Cd45$^+$ cells) sorting from the spinal cord of mice following EAE induction. **b-d** Quantification of microglia (MG), macrophages, monocytes and Cd11c$^+$ MG (**b**); B cells, Neutrophils, Cd4$^+$ T cells and Cd8$^+$ T cells (**c**); total cells and Cd45$^+$ cells (**d**) in the spinal cord of female WT and p16 KO mice following EAE induction. Mice were sacrificed during the acute phase of EAE (on day 22). **e-g** Quantification of macrophages and monocytes (**e**); B cells, Neutrophils, Cd4$^+$ T cells and Cd8$^+$ T cells (**f**); total cells and Cd45$^+$ cells (**g**) in the spleen of female WT and p16 KO mice following EAE induction. Mice were sacrificed in the acute phase of EAE (on day 22). The cell counts were normalised as the number of cells in the whole spinal cord or spleen per mouse. **h-k** Proportion of each cell type in Cd45$^+$ cells in the spinal cord (**h-i**) and spleen (**j-k**) of female WT and p16 KO mice following EAE induction, related to (**b-g**). Data presented as mean ± S.E.M from four mice. Statistical significance was determined with two-tailed unpaired Student's t-test (**b-g**). n.s., non-significant.

Supplementary Fig. 11

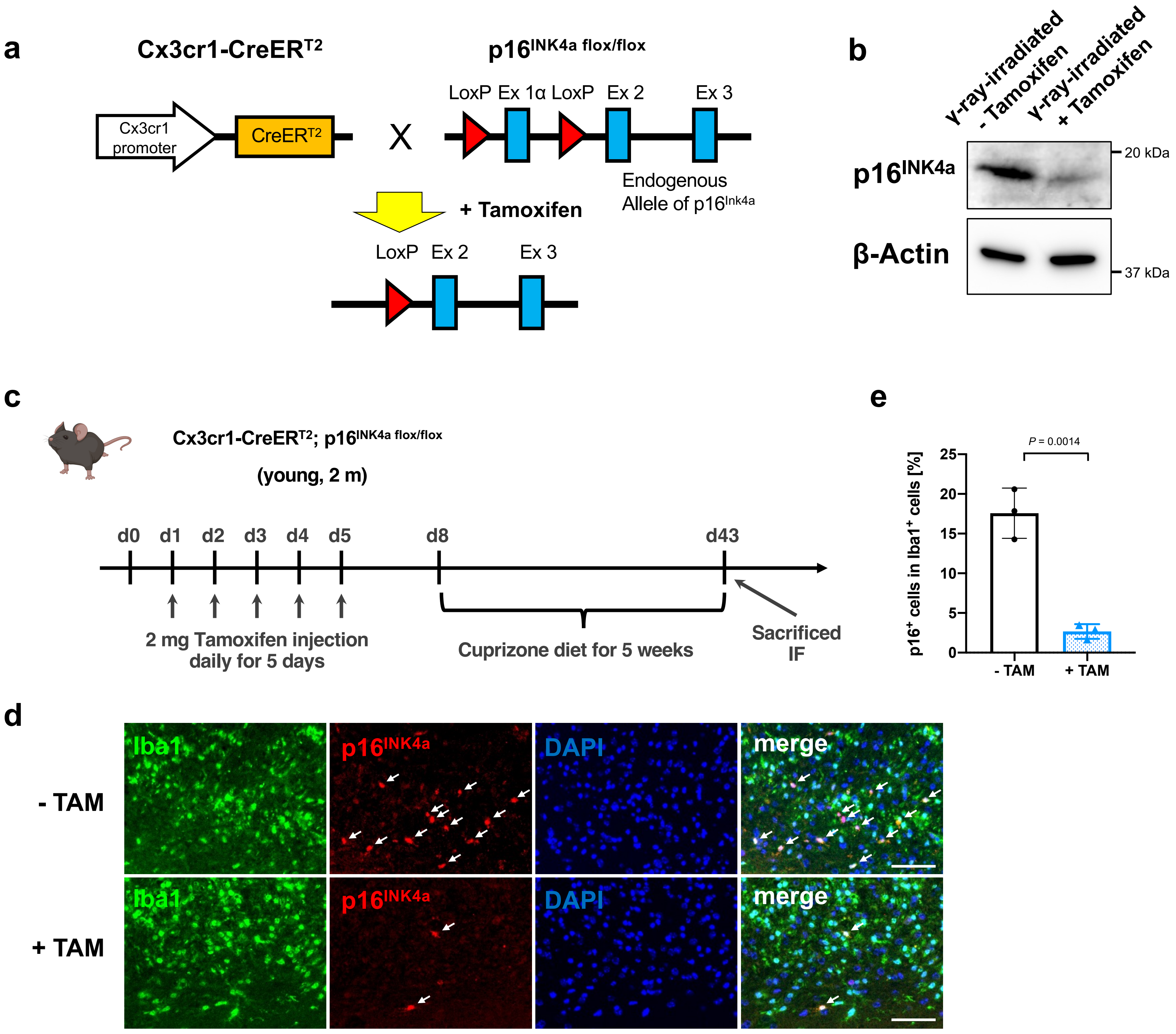

Supplementary Fig. 11
Evaluation of Cx3cr1-CreER^{T2}: p16$^{flox/flox}$ mice.
a Schematic depicting generation of Cx3cr1-CreER^{T2}: p16$^{flox/flox}$ mice. **b**, young (3 months) male Cx3cr1-CreER$^{T2;}$ p16$^{flox/flox}$ mice were treated daily for five days with corn oil with or without tamoxifen and then irradiated with γ-radiation at 10 Gy. The mice were sacrificed 3 months after irradiation and lysates from Cd11b^{+} cells isolated from the brain via MACS were immunoblotted for p16^{INK4a} protein. β-Actin was used as a loading control. **c** Overview of tamoxifen (TAM) treatment and feeding of mice with cuprizone-containing diet in young (2 months) male Cx3cr1-CreER^{T2}: p16$^{flox/flox}$ mice. Mice were intraperitoneally injected with tamoxifen daily for 5 days (on d1 to d5) and fed with cuprizone diet for five weeks (on d8 to d43). Mice were then sacrificed on d43. The figure was created with BioRender.com. **d** Representative immunofluorescence images for Iba1 (green), p16^{INK4a} (red), and DAPI (blue) in the corpus callosum of young (2 months) male Cx3cr1-CreER^{T2}: p16$^{flox/flox}$ mice injected with corn oil with or without tamoxifen were fed with cuprizone diet for 5 weeks. Iba1/p16^{INK4a}-positive cells are indicated by arrows. **e** Quantification of p16^{INK4a} expression amongst Iba1^{+} cells in (**d**) (-TAM: N = 3, n = 213 - 238 Iba1^{+} cells; +TAM: N = 3, n = 231 - 243 Iba1^{+} cells per mouse). Data presented as mean ± S.E.M. Statistical significance was determined with two-tailed unpaired Student's t-test (**e**). Scale bar, 50 µm.

Supplementary Fig. 12

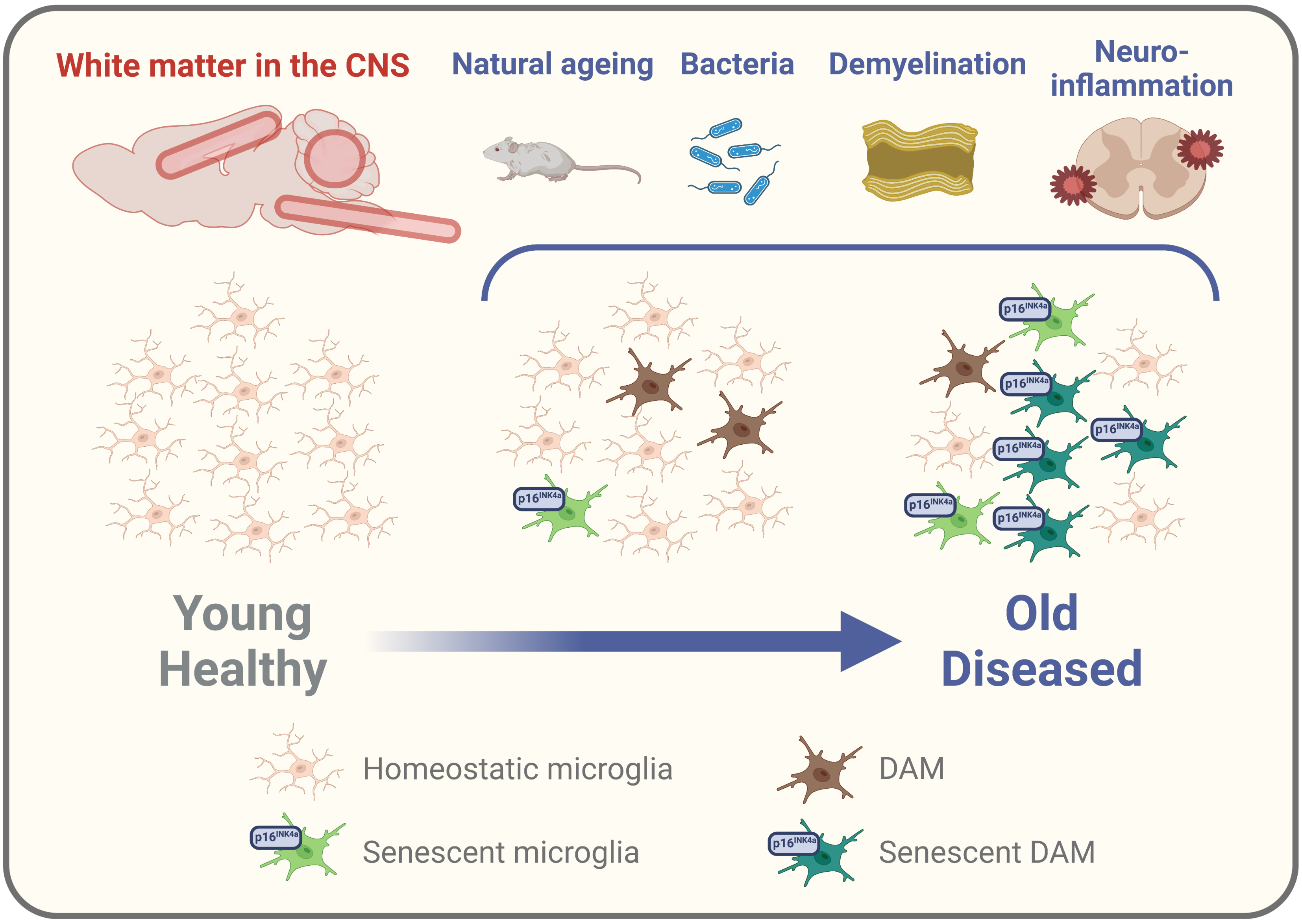

Supplementary Fig. 12
The regulation of cellular senescence in microglia in the CNS under ageing and diseased conditions.
A model of the regulation of cellular senescence in homeostatic microglia and DAM in the white matter by natural ageing, bacteria, demyelination and neuroinflammation. The figure was created with BioRender.com.

Supplementary Fig. 13

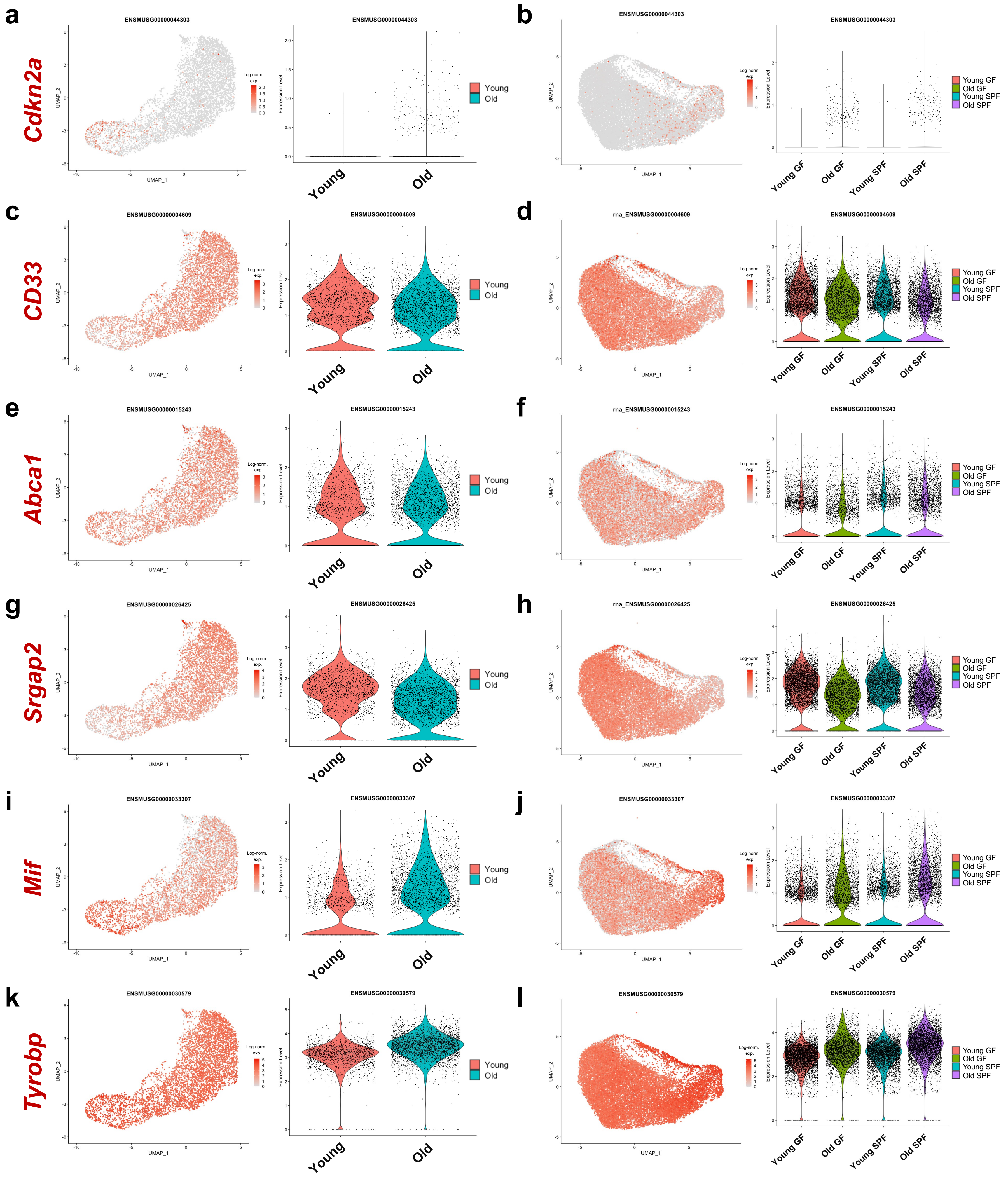

Supplementary Fig. 13

WAM- or DAM-specific gene expression in the white matter of young and old mice.

a-l UMAP plots depicting the expression patterns or graphs showing expression level of *Cdkn2a* (**a-b**), WAM-specific genes *Cd33* (**c-d**), *Abca1* (**e-f**) and *Srgap2* (**g-h**) and DAM-specific genes *Mif* (**i-j**) and *Tyrobp* (**k-l**) in microglia from the corpus callosum of male young/old mice (**a,c,e,g,i,k**) or medulla oblongata of male young/old GF/SPF mice (**b,d,f,h,j,l**). Log-norm. exp., Log-normalised expression.

Supplementary Fig. 14

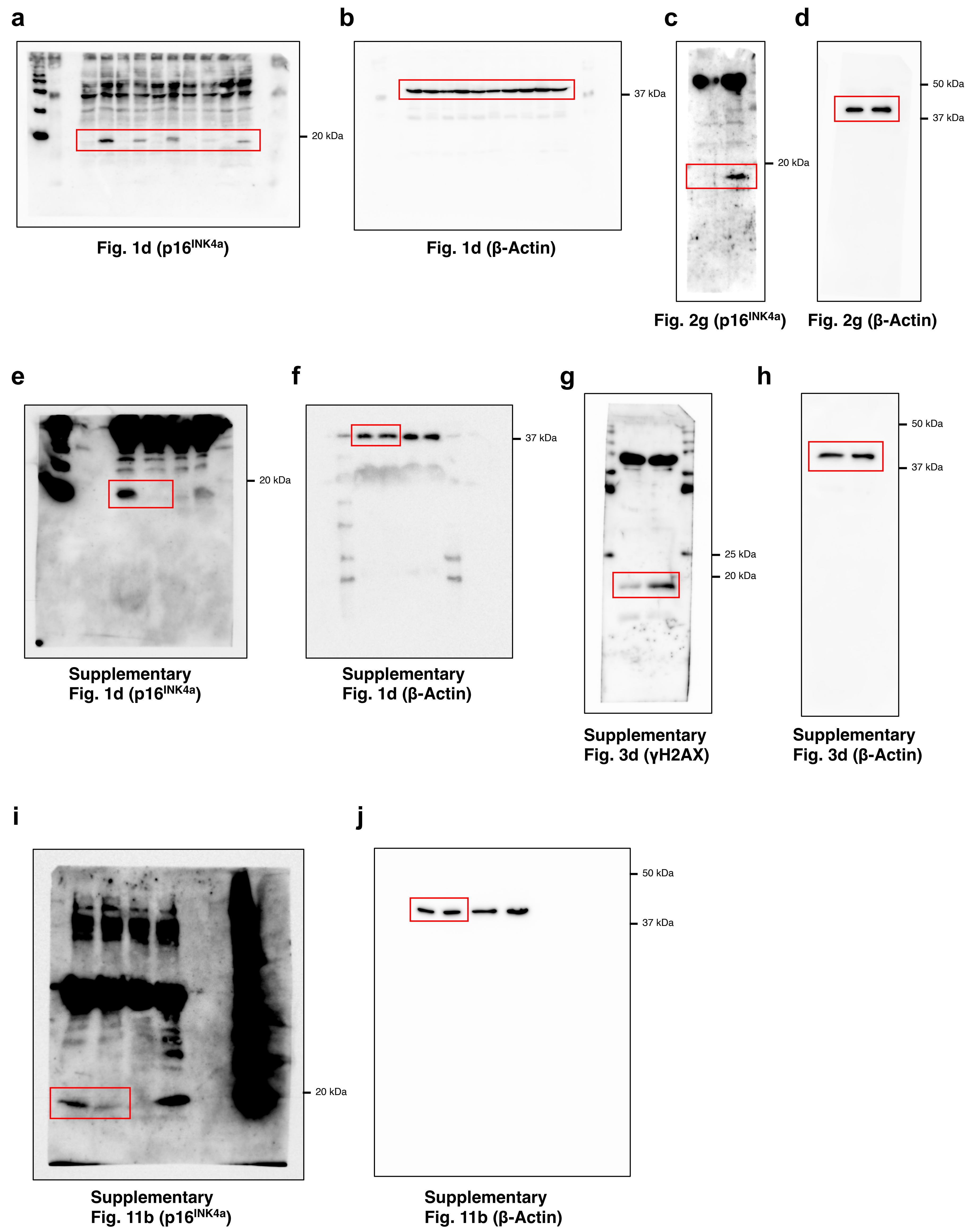

Supplementary Fig. 14
Original and uncropped images for the immunoblotting experiment shown in this study.
