# Supplementary material for: Cellular senescence in white matter microglia is induced during ageing in mice and exacerbates the neuroinflammatory phenotype
Source: Commun Biol. 2023 Jun 23;6:665. doi: 10.1038/s42003-023-05027-2 (PMC10290132; doi:10.1038/s42003-023-05027-2)
Supplement: Supplementary file 5 — Reporting Summary [file 42003_2023_5027_MOESM5_ESM.pdf]

nature portfolio

Corresponding author(s): Tatsuyuki Matsudaira

Last updated by author(s): Jun 6, 2023

Reporting Summary

Nature Portfolio wishes to improve the reproducibility of the work that we publish. This form provides structure for consistency and transparency in reporting. For further information on Nature Portfolio policies, see our Editorial Policies and the Editorial Policy Checklist.

Statistics

For all statistical analyses, confirm that the following items are present in the figure legend, table legend, main text, or Methods section.

n/a	Confirmed	
☐	☒	The exact sample size (*n*) for each experimental group/condition, given as a discrete number and unit of measurement
☐	☒	A statement on whether measurements were taken from distinct samples or whether the same sample was measured repeatedly
☐	☒	The statistical test(s) used AND whether they are one- or two-sided<br>*Only common tests should be described solely by name; describe more complex techniques in the Methods section.*
☒	☐	A description of all covariates tested
☐	☒	A description of any assumptions or corrections, such as tests of normality and adjustment for multiple comparisons
☐	☒	A full description of the statistical parameters including central tendency (e.g. means) or other basic estimates (e.g. regression coefficient) AND variation (e.g. standard deviation) or associated estimates of uncertainty (e.g. confidence intervals)
☐	☒	For null hypothesis testing, the test statistic (e.g. *F*, *t*, *r*) with confidence intervals, effect sizes, degrees of freedom and *P* value noted<br>*Give P values as exact values whenever suitable.*
☒	☐	For Bayesian analysis, information on the choice of priors and Markov chain Monte Carlo settings
☒	☐	For hierarchical and complex designs, identification of the appropriate level for tests and full reporting of outcomes
☒	☐	Estimates of effect sizes (e.g. Cohen's *d*, Pearson's *r*), indicating how they were calculated

Our web collection on statistics for biologists contains articles on many of the points above.

Software and code

Policy information about availability of computer code

Data collection	Single cell RNA-seq analysis : Illumina NovaSeq 6000 sequencer (Illumina)<br>Bulk RNA-seq analysis: Illumina NovaSeq 6000 sequencer (Illumina)<br>Microscopic data: Fluorescence Microscope BZ-X710 and BZ-X800 (KEYENCE)<br>Quantitative real-time PCR data: Thermal Cycler Dice Real Time System III TP970 (TAKARA Bio Inc.)<br>Immunoblotting data: LAS-3000mini imaging system (Fujifilm)<br>RNA sequencing data: Illumina HiSeq 2500 platform (Illumina)<br>Histological data: BX53 Upright Microscope (OLYMPUS)<br>Flow cytometry: SONY SH800 Sorter (SONY) and Attune Nxt Autosampler (ThermoFisher Scientific)<br>Bioluminescence imaging: Living Image Software 4.7.3 (Perkin Elmer)
Data analysis	Single cell and bulk RNA sequencing analysis: data analysis was performed with open source software as described in the methods of the paper<br>Immunoblotting analysis: Fiji (ImageJ)<br>Immunofluorescence staining analysis: Fiijii (ImageJ) and BZ-X analyzer software (Keyence)<br>Histological data: cellSens Standard (OLYMPUS)<br>Bioluminescence imaging: IVIS Lumina XRMS Series III (Perkin Elmer)<br>Flow cytometry: FlowJo v10.8.1<br>Statistical analysis: Prism 8 and R

For manuscripts utilizing custom algorithms or software that are central to the research but not yet described in published literature, software must be made available to editors and reviewers. We strongly encourage code deposition in a community repository (e.g. GitHub). See the Nature Portfolio guidelines for submitting code & software for further information.

Data

Policy information about availability of data

All manuscripts must include a data availability statement. This statement should provide the following information, where applicable:

- Accession codes, unique identifiers, or web links for publicly available datasets
- A description of any restrictions on data availability
- For clinical datasets or third party data, please ensure that the statement adheres to our policy

ScRNA-seq analysis (DNA Data Bank of Japan (DDBJ), DRA014178) and bulk RNA-seq analysis (Gene Expression Omnibus database (GEO), GSE204828)

Human research participants

Policy information about studies involving human research participants and Sex and Gender in Research.

Reporting on sex and gender	*Use the terms sex (biological attribute) and gender (shaped by social and cultural circumstances) carefully in order to avoid confusing both terms. Indicate if findings apply to only one sex or gender; describe whether sex and gender were considered in study design whether sex and/or gender was determined based on self-reporting or assigned and methods used. Provide in the source data disaggregated sex and gender data where this information has been collected, and consent has been obtained for sharing of individual-level data; provide overall numbers in this Reporting Summary. Please state if this information has not been collected. Report sex- and gender-based analyses where performed, justify reasons for lack of sex- and gender-based analysis.*
Population characteristics	*Describe the covariate-relevant population characteristics of the human research participants (e.g. age, genotypic information, past and current diagnosis and treatment categories). If you filled out the behavioural & social sciences study design questions and have nothing to add here, write "See above."*
Recruitment	*Describe how participants were recruited. Outline any potential self-selection bias or other biases that may be present and how these are likely to impact results.*
Ethics oversight	*Identify the organization(s) that approved the study protocol.*

Note that full information on the approval of the study protocol must also be provided in the manuscript.

Field-specific reporting

Please select the one below that is the best fit for your research. If you are not sure, read the appropriate sections before making your selection.

☒ Life sciences ☐ Behavioural & social sciences ☐ Ecological, evolutionary & environmental sciences

For a reference copy of the document with all sections, see nature.com/documents/nr-reporting-summary-flat.pdf

Life sciences study design

All studies must disclose on these points even when the disclosure is negative.

Sample size	The sample size used in this study was determined due to the expense of data collection and the need for sufficient statistical power.
Data exclusions	*Describe any data exclusions. If no data were excluded from the analyses, state so OR if data were excluded, describe the exclusions and the rationale behind them, indicating whether exclusion criteria were pre-established.*
Replication	All data shown are from twotindependent experiments with similar results. (see Figure legend)
Randomization	Mice were randomized to receive treatments.
Blinding	*Describe whether the investigators were blinded to group allocation during data collection and/or analysis. If blinding was not possible, describe why OR explain why blinding was not relevant to your study.*

Reporting for specific materials, systems and methods

We require information from authors about some types of materials, experimental systems and methods used in many studies. Here, indicate whether each material, system or method listed is relevant to your study. If you are not sure if a list item applies to your research, read the appropriate section before selecting a response.

Materials & experimental systems

n/a	Involved in the study
☐	☒ Antibodies
☒	☐ Eukaryotic cell lines
☒	☐ Palaeontology and archaeology
☐	☒ Animals and other organisms
☒	☐ Clinical data
☒	☐ Dual use research of concern

Methods

n/a	Involved in the study
☒	☐ ChIP-seq
☐	☒ Flow cytometry
☒	☐ MRI-based neuroimaging

Antibodies

Antibodies used

For flow cytometry,
CD16/32 FcR-blocking reagent (cat#: 101330, clone: 93), FITC anti-Cd11b (cat#: 101206, clone: M1/70), FITC-conjugated anti-Cd45 (cat#: 103108, clone: 30-F11), PE-conjugated anti-Cd11c (cat#: 117307, clone: N418), PE-conjugated anti-Cd8a (cat#: 100708, clone: 53–6.7), PerCP/Cy5.5-conjugated anti-Cd11b (cat#: 101228, clone: M1/70), PerCP/Cy5.5-conjucated anti-Cd45 (cat#: 103132, clone: 30-F11), PE/Cy7-conjugated anti-Cd45 (cat#: 103114, clone: 30-F11), PE/Cy7-conjugated anti-F4/80 (cat#: 123114, clone: BM8), PE/Cy7-conjugated anti-Cd4 (cat# 100528, clone: RM4–5), APC-conjugated anti-Cd11b (cat#: 101212, clone: M1/70), APC-conjugated anti-Cx3cr1 (cat#: 149008, clone: SA011F11), APC-conjugated anti-CD3ε (cat#: 100312, clone: 145–2C11), Brilliant Violet 421™-conjugated anti-CD45R/B220 (cat#: 103240, clone: RA3–6B2), Brilliant Violet 421™-conjugated anti-Ly6g (cat#: 127628, clone: 1A8).

For immunoblotting,
β-actin (1:10000, Merck Millipore, cat#: A5316), p16INK4a (1:2000, abcam, UK, cat#: ab211542), γH2AX (1:1000, abcam, cat#: ab2893) and secondary antibodies (1:2000, Cell signaling Technology, MA, USA).

For immunostaining,
rabbit anti-p16 (1:200, cat#: ab211542, abcam), rabbit anti-Iba1 (1:1000, Fujifilm, cat#: 019-19741), guinea pig anti-Iba1 (1:1000, Synaptic Systems, Germany, cat#: 234004, 234308), rat anti-Lgals3 (1:3000, Biolegend, cat#: 125401), Alexa Fluor Plus 488-conjugated donkey anti-rabbit IgG (1:2000; ThermoFisher Scientific, cat# A32790), Alexa Fluor Plus 488-conjugated donkey anti-guinea pig IgG (1:2000; JacksonImmunoResearch, UK, cat#: 706-545-148), Alexa Fluor Plus 555-conjugated donkey anti-rabbit IgG (1:2000; ThermoFisher Scientific, cat#: A32794) and Alexa Fluor Plus 647-conjugated donkey anti-rat IgG (1:2000; ThermoFisher Scientific, cat#: A48272).

Validation

All antibodies used in this study were commercially available antibodies and were validated by the companies.

Antibodies for flow cytometry were purchased from Biolegend.
CD16/32 FcR-blocking reagent (cat#: 101330, clone: 93)
FITC anti-Cd11b (cat#: 101206, clone: M1/70)
FITC-conjugated anti-Cd45 (cat#: 103108, clone: 30-F11),
PE-conjugated anti-Cd11c (cat#: 117307, clone: N418),
PE-conjugated anti-Cd8a (cat#: 100708, clone: 53–6.7),
PerCP/Cy5.5-conjugated anti-Cd11b (cat#: 101228, clone: M1/70),
PerCP/Cy5.5-conjucated anti-Cd45 (cat#: 103132, clone: 30-F11),
PE/Cy7-conjugated anti-Cd45 (cat#: 103114, clone: 30-F11),
PE/Cy7-conjugated anti-F4/80 (cat#: 123114, clone: BM8),
PE/Cy7-conjugated anti-Cd4 (cat# 100528, clone: RM4–5),
APC-conjugated anti-Cd11b (cat#: 101212, clone: M1/70),
APC-conjugated anti-Cx3cr1 (cat#: 149008, clone: SA011F11),
APC-conjugated anti-CD3ε (cat#: 100312, clone: 145–2C11),
Brilliant Violet 421™-conjugated anti-CD45R/B220 (cat#: 103240, clone: RA3–6B2),
Brilliant Violet 421™-conjugated anti-Ly6g (cat#: 127628, clone: 1A8).

For immunoblotting,
β-actin (1:10000, Merck Millipore, cat#: A5316): https://www.sigmaaldrich.com/JP/ja/product/sigma/a5316
p16INK4a (1:2000, abcam, UK, cat#: ab211542): https://www.abcam.com/cdkn2ap16ink4a-antibody-epr20418-ab211542.html
γH2AX (1:1000, abcam, cat#: ab2893): https://www.abcam.com/gamma-h2ax-phospho-s139-antibody-ab2893.html

For immunostaining,
rabbit anti-p16 (1:200, cat#: ab211542, abcam): https://www.abcam.com/cdkn2ap16ink4a-antibody-epr20418-ab211542.html
rabbit anti-Iba1 (1:1000, Fujifilm, cat#: 019-19741): https://labchem-wako.fujifilm.com/us/product/detail/W01W0101-1974.html
guinea pig anti-Iba1 (1:1000, Synaptic Systems, Germany, cat#: 234004): https://sysy.com/product/234308
(This Product (cat# 234004) has been discontinued, it has been replaced by 234308.)
rat anti-Lgals3 (1:3000, Biolegend, cat#: 125401): https://www.biolegend.com/en-us/products/purified-anti-mouse-human-mac-2-galectin-3-antibody-4935?GroupID=BLG2786

Animals and other research organisms

Policy information about studies involving animals; ARRIVE guidelines recommended for reporting animal research, and Sex and Gender in Research

Laboratory animals	C57BL/6J mice, Albino p16-luc mice, p16 knockout mice, Cx3cr1-CreERT2 mice, p16flox/flox mice and SOD1 G93A mice.
Wild animals	No wild animals were used in the study.
Reporting on sex	The p16-luc mice and the EAE-induced mice were female. The other mice were male.
Field-collected samples	The study did not involve samples collected from the field.
Ethics oversight	All mouse experiments were approved by the Animal Research Committee of the Research Institute for Microbial Diseases at Osaka University.

Note that full information on the approval of the study protocol must also be provided in the manuscript.

Flow Cytometry

Plots

Confirm that:

☒ The axis labels state the marker and fluorochrome used (e.g. CD4-FITC).

☒ The axis scales are clearly visible. Include numbers along axes only for bottom left plot of group (a 'group' is an analysis of identical markers).

☒ All plots are contour plots with outliers or pseudocolor plots.

☒ A numerical value for number of cells or percentage (with statistics) is provided.

Methodology

Sample preparation	The method for sample preparation of each tissue is described in detail in the method section.
Instrument	SONY SH800 Sorter (SONY, Japan) and Attune Nxt Autosampler (ThermoFisher Scientific, MA, USA)
Software	FlowJo v10.8.1
Cell population abundance	The post-sorted fraction analysed after the Cd11c+ or Cd11c- MG fraction was not abundant due to the limited number of cells.
Gating strategy	For FSC/SSC gating, the fraction in which most microglial cells or other cell types have been shown to accumulate by backgating was used.

☒ Tick this box to confirm that a figure exemplifying the gating strategy is provided in the Supplementary Information.
